# Innovative Approach in Nursing Care: Artificial Intelligence-Assisted Incentive Spirometry

**DOI:** 10.3390/healthcare13212693

**Published:** 2025-10-24

**Authors:** Yusuf Uzun, İbrahim Çetin, Mehmet Kayrıcı

**Affiliations:** 1 Department of Computer Engineering, Faculty of Seydişehir Ahmet Cengiz Engineering, Necmettin Erbakan University, Konya 42370, Türkiye; 2 Department of Nursing, Faculty of Seydisehir Kamil Akkanat Health Sciences, Necmettin Erbakan University, Konya 42370, Türkiye; ibrahim.cetin@erbakan.edu.tr; 3 Department of Mechanical Engineering, Faculty of Seydişehir Ahmet Cengiz Engineering, Necmettin Erbakan University, Konya 42370, Türkiye; mkayrici@erbakan.edu.tr

**Keywords:** artificial intelligence, incentive spirometry, breathing exercise, machine learning, image processing

## Abstract

**Background/Objectives:** This study presents an artificial intelligence (AI)-supported incentive spirometry system designed to explore the feasibility of automating the monitoring of respiratory exercises, a critical nursing intervention for maintaining pulmonary function and reducing postoperative complications. **Methods:** This system uses a tablet’s camera to track a standard spirometer’s volume indicator in real-time, reducing the manual nursing workload, unlike traditional mechanical spirometers that lack feedback capabilities. Image processing techniques analyze exercise performance, while the interface provides instant feedback, data recording, and graphical display. Machine learning models (Random Forest, XGBoost, Gradient Boosting, SVM, Logistic Regression, KNN) were trained on scripted patient data, including demographics, smoking status, and spirometry measurements, to classify respiratory performance as “poor”, “good”, or “excellent”. **Results:** The ensemble methods demonstrated exceptional performance, achieving 100% accuracy and R^2^ = 1.0, with cross-validation mean accuracies exceeding 99.4%. This feasibility study demonstrates the technical viability of this AI-driven approach and lays the groundwork for future clinical validation. **Conclusions:** This system presents a potential cost-effective, accessible solution suitable for both clinical and home settings, potentially integrating into standard respiratory care protocols. This system not only reduces nursing workload but also has the potential to improve patient adherence. This pilot study demonstrates the technical feasibility and potential of this AI-driven approach, laying the groundwork for future clinical validation.

## 1. Introduction

Physiologically, ventilation, cardiac output, and pulmonary perfusion mechanisms must work synchronously to meet the increased oxygen demand during exercise. Lung diseases limit this physiological response, reducing activity capacity. Therefore, maintaining and even improving activity capacity is crucial for preserving patients’ pulmonary function [1,2].

Surgeries, like lung diseases, make it difficult to maintain pulmonary functions and lead to complications. Postoperative pulmonary complications are common problems that negatively impact patient health and treatment processes. These complications can lead to serious consequences such as atelectasis, pneumonia, and collapse. Therefore, supporting pulmonary function with effective breathing exercises is crucial [3]. Traditionally used mechanical incentive spirometers require one-on-one physical attention and time from clinical nurses during exercise. Furthermore, the mechanical devices currently in use lack features such as data recording, analysis, and feedback. These deficiencies limit the effective and efficient implementation of exercise and the evaluation of exercise performance [4].

Postoperative pulmonary complications are among the most important factors increasing surgical morbidity and mortality. Atelectasis, secretion retention, and impaired respiratory mechanics, particularly after thoracic, cardiac, and upper abdominal surgeries, can negatively affect postoperative recovery and lead to a significant decrease in respiratory function, as well as a prolonged hospital stay. Therefore, physiologically based breathing exercises have become an important preventive approach in the postoperative period [2,5]. In this context, incentive spirometry encourages the user to take deep and slow inspirations, increasing lung ventilation and reducing alveolar collapse. However, most studies in the literature offer limited and conflicting results regarding the effectiveness of existing incentive spirometers; the vast majority of devices used provide only mechanical feedback and do not produce meaningful data regarding the exercise process [6,7]. Furthermore, this dependence on one-on-one supervision creates significant inefficiencies. Studies indicate that nurses spend an average of only 10-15 min per patient per shift on specific prophylactic interventions, such as spirometry monitoring, due to high patient-to-nurse ratios and competing clinical demands [4]. This time pressure, combined with reports that up to 40% of nurses lack formal training in incentive spirometry protocols, severely limits the effectiveness of these exercises [8]. The developed AI system directly addresses this burden by automating the monitoring process, potentially freeing up valuable nursing time for other critical care activities.

An incentive spirometer is a mechanical device widely used to promote deep and controlled breathing, thereby increasing alveolar ventilation and reducing the risk of postoperative complications. However, most incentive spirometer devices currently used in clinics offer only mechanical flow feedback. They lack the capacity to generate and analyze user data or communicate information to healthcare professionals. Furthermore, studies have shown that a large portion of both nurses and patients lack knowledge about the correct use patterns and methods of IS devices. Therefore, findings regarding the effectiveness of exercises can be contradictory [3,8].

In recent years, artificial intelligence (AI) technologies have been effectively used in a wide variety of healthcare areas, from clinical decision support systems to patient monitoring and education. This study specifically leverages machine learning (ML), a subset of AI that enables systems to learn and make predictions from data without being explicitly programmed for every rule. It is important to distinguish this from generative AI, which creates new content; our work focuses on discriminative and predictive AI for analysis and classification. ML-supported systems facilitate behavioral change by providing personalized feedback to users and offering healthcare professionals data-driven solutions for informed decision-making [9].

Advances in medical technologies offer innovative solutions for automatically monitoring respiratory exercises. Image processing and artificial intelligence-based systems have the potential to objectively and in real time assess patients’ respiratory performance [10]. Deep learning algorithms, in particular, have achieved high accuracy in analyzing spirometric data [11]. Similarly, mobile health applications allow patients to self-monitor at home or in clinical settings [12].

However, most existing solutions require expensive sensors or complex hardware, limiting their widespread use [13]. In this context, the need arises to develop a low-cost and accessible system. Image processing-based approaches allow for accurate tracking of spirometric measurements using a standard tablet camera [14]. Furthermore, machine learning models can analyze patient data and provide personalized feedback [10].

Table 1 presents a comparative analysis of AI and digital spirometry approaches. While existing solutions offer portability or clinical decision support, they often rely on additional hardware or specialized sensors, which can increase costs and limit accessibility. In contrast, the system proposed in this study utilizes a standard tablet camera and powerful image processing algorithms, providing a low-cost, software-based solution that does not require any additional hardware. The developed system is designed for use in both hospital and home environments. By combining real-time image processing, machine learning classification, and a user-friendly interface within a single platform, it provides a solution that enhances nursing care and supports patient management.

The literature comparison of AI/digital spirometry approaches and the contribution of this study are shown in Table 1.

The effectiveness of incentive spirometry depends on accurate and consistent application. This has traditionally required direct or one-on-one visual supervision by nurses to achieve correct technique [4,8]. This manual monitoring model is very difficult to maintain in modern healthcare settings characterized by high nurse-to-patient ratios and clinical demands. It creates a significant workload for nurses and limits the frequency and quality of supervision, ultimately compromising patient outcomes. This study addresses this gap by developing an AI-powered system that automates the supervision and feedback process. This system aims to augment the nurse’s oversight role (ensuring correct execution of exercises) while also optimally utilizing nursing time for other critical tasks, potentially improving both care efficiency and patient compliance.

This study aims to develop an artificial intelligence-supported incentive spirometry system that utilizes image processing to augment nursing care and enhance patient adherence to respiratory exercises. The system monitors spirometric movements using a standard tablet camera for real-time analysis and classifies respiratory performance using machine learning models. This aims to reduce clinician workload and more effectively manage patients’ respiratory exercise processes. Despite the increasing use of AI in respiratory care, no previous study has integrated real-time image processing with incentive spirometry for both hospital and home-based nursing applications. This study addresses this gap.

## 2. Materials and Methods

**Figure 1:**
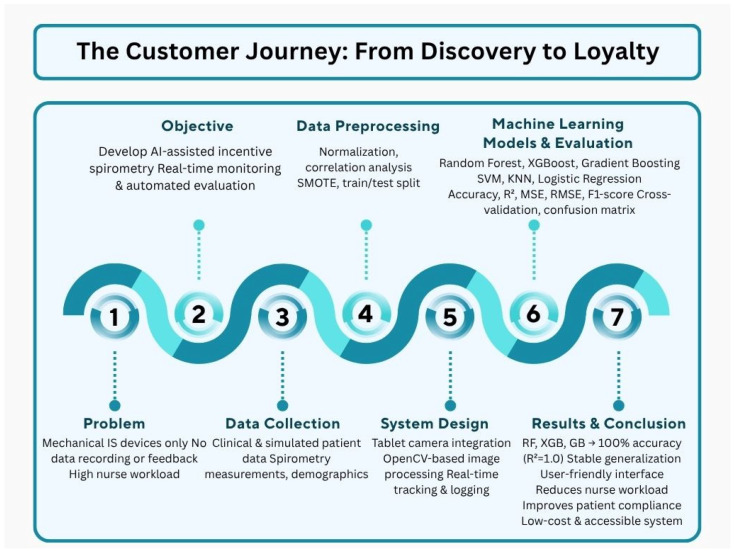
Flowchart diagram of the AI-assisted incentive spirometry system and methodological framework.

The schematic diagram illustrating the comprehensive flowchart of the study is shown in Figure 1. Starting from the limitations of mechanical spirometers, this approach presents an integrated solution covering data collection, preprocessing, machine learning models, and result evaluation. The developed system aims to reduce nursing workload while improving patient compliance and providing a low-cost solution.

### 2.1. Dataset and Data Preprocessing

The dataset used in the study included clinical data and spirometric measurements from 250 patients based on simulated scenarios. It is important to note that this dataset was simulated and not collected from real patients. The simulated data were generated based on clinical parameters and spirometry patterns observed in standard postoperative care protocols. Feature distributions (e.g., age, spirometry measurements) were designed to mimic real-world patient cohorts to ensure the ecological validity of the machine learning models trained on this data. The simulated dataset, consisting of 250 scripted patient scenarios, was designed to replicate clinical variability in postoperative respiratory care, including demographics, smoking status, and spirometry measurements at timed intervals. No real patient data was used as a direct source, and no human subjects were involved in data generation. Descriptive statistics (Table 2) reveal a mean age of 55.04 ± 24.26 years (range: 12–94), with spirometry values showing slight left skewness indicative of higher performance trends (e.g., average spirometry: 2420.20 ± 638.73 mL, range: 1000–3500). Categorical breakdowns highlight a balanced distribution across disease categories (e.g., 19.2% each for internal medicine adult and surgery adult) and a predominance of non-smokers (65.2%), while the respiratory performance classes exhibit imbalance (63.6% “beautiful”, 36.0% “perfect”, 0.4% “low”), which was mitigated through SMOTE augmentation during preprocessing. This summary underscores the dataset’s ecological validity for training robust machine learning models. The data were structured as follows: treatment type (medical-surgical), age, smoking status, and cross-sectional spirometry measurements at 30, 60, 90, and 120 s, as well as mean respiratory volume and respiratory performance classification (“poor”, “good”, and “excellent”). Similar studies indicate that such clinical datasets are widely used in machine learning applications [16].

The original class distribution exhibited significant imbalance for the ‘low’ performance class, which contained a single sample (Table 2). Such an imbalance can bias machine learning models toward the majority classes (“nice” and “perfect”). This can mitigate the problem of generalizing to classes that are underrepresented in real-world settings. To mitigate this, the SMOTE algorithm was used to oversample the minority classes synthetically. The inherent limitations of this approach, particularly for the lower class, were acknowledged. Generating a specific number of synthetic samples from a single data point introduces the risk of creating artifacts that may not accurately reflect the true variance within that class. Consequently, the SMOTE model’s ability to recognize the possibility of a ‘low’ performance classification is improved. At the same time, its generalizability to a specific class across a diverse clinical population provides a model for future validation with a larger, real-world patient dataset. The primary goal of data augmentation was to prevent the model from completely ignoring these classes, rather than perfectly modeling the complex features of the minority classes.

To improve the performance of machine learning models, numerical variables (such as age and spirometry measurements) were scaled to the range [0, 1] using the Min-Max normalization method. Pearson correlation analysis was applied to check for multicollinearity between features, and highly correlated data (|r| > 0.8) was removed from model training [17]. To reliably assess model performance, the dataset was randomly split into 80% training and 20% test. To ensure a realistic performance evaluation while preventing data leakage, all data preprocessing steps, including the application of the SMOTE (Synthetic Minority Oversampling Technique) algorithm to address class distribution imbalances, were applied only to the training set. A 10-fold cross-validation method was used to prevent overfitting. Data augmentation was performed using the SMOTE (Synthetic Minority Over-sampling Technique) algorithm to address class distribution imbalances [18].

### 2.2. Data Recording and Analysis

In this study, a real-time data recording and analysis process was designed to determine the levels of a white, moving object on the spirometer device (as shown in the system flowchart, Figure 1). This process enables the evaluation of user performance by processing, recording, and analyzing the information obtained from the device. The real-time system simultaneously displayed the detected respiratory volume levels on the screen and recorded them in a log file. Each record includes a timestamp, the level reached, and the maximum object movement level. This structure enables the chronological analysis of data and the comparison of user performance with records. The data was stored in CSV (Comma-Separated Values) format for easy access and analysis.

The recorded data was analyzed to evaluate the user’s respiratory exercise performance. The data in the log file was visualized as charts using the matplotlib library. These charts clearly show the maximum levels reached by the user and performance trends. Basic statistical calculations, including mean, median, and standard deviation, were performed on the recorded data. These analyses provided predictions of the user’s long-term respiratory performance. Error-checking mechanisms were implemented during real-time data processing to ensure the accuracy and integrity of recorded data. Data was stored in a secure environment against external interference. This structured data recording and analysis process provides an effective infrastructure for detailed user performance monitoring, enabling usable predictions for clinical decision support systems.

### 2.3. Incentive Spirometer

**Figure 2:**
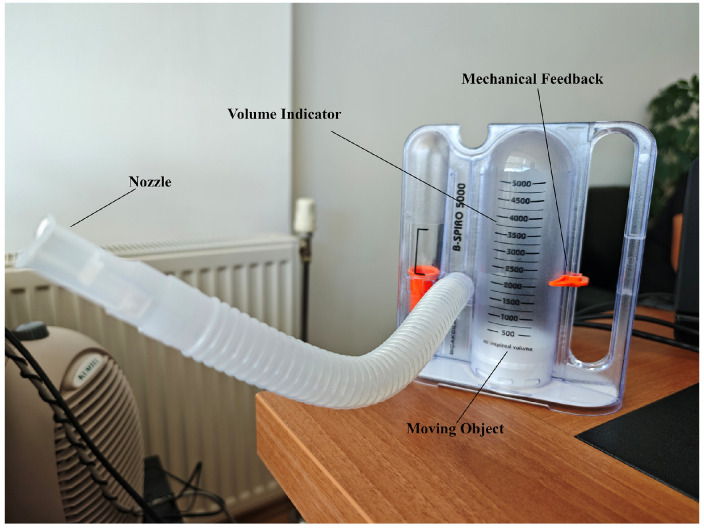
Incentive Spirometer.

In this study, a real-time data recording and analysis process was designed to determine the levels of a white, moving object on a spirometer device (Figure 2: moving object component). This process enables the evaluation of user performance by processing, recording, and analyzing the information obtained from the device. The real-time system simultaneously displayed the detected respiratory volume levels on the screen and recorded them in a log file. Each record includes a timestamp, the level reached, and the maximum object movement level. This structure enables the chronological analysis of data and the comparison of user performance with records. The data was stored in CSV (Comma-Separated Values) format for easy access and analysis.

A volumetric incentive spirometer capable of high-precision measurements in the range of 0–5000 mL was used in the study (Figure 2). The device’s main technical features are as follows:

- Measurement range: 0–5000 mL (±3% accuracy)
- Volume display: Milliliter-based scale reading with a transparent cylindrical chamber
- Flow rate measurement: 100–1200 mL/s
- Integration of a disposable mouthpiece and bacterial filter
- Mechanical feedback system: Movable marker for patients to visually monitor the target volume [19].

The device’s metrological validity was tested in accordance with ISO 26782:2009 standards [20]:

- Volume accuracy: 98.2% correlation (*p* < 0.01) in the 500–5000 mL range
- Repeatability: ±1.8% CV (Coefficient of Variation) in 20 measurements
- Clinical validation: 0.95 ICC (Intraclass Correlation) agreement with a standard spirometer in a comparative study on 50 patients [19].

### 2.4. Image Processing

The OpenCV (Open Source Computer Vision Library) 4.7.0 library was used for image processing. The algorithm developed for spirometer piston detection and tracking consisted of four main stages in a real-time video stream [21].

In the first stage, the 1280 × 720 resolution, 30 fps video stream captured from the tablet camera was subjected to noise reduction using Gaussian filtering (σ = 1.5). Histogram equalization was applied for image stabilization, and contrast enhancement was performed [22]. For color space conversion, a robust infrastructure against illumination changes was created by switching from BGR to HSV space [23].

In the object detection stage, an adaptive thresholding method was used to determine the position of the spirometer piston. A binary image was obtained using Otsu’s thresholding algorithm, and noise was reduced using morphological operations (erosion and dilation) [24]. Contour analysis enabled the determination of piston boundaries and the calculation of bounding box coordinates.

The Kanade-Lucas-Tomasi (KLT) feature tracking algorithm was implemented for motion tracking. Optical flow calculations were performed using the Lucas-Kanade method using a pyramidal approximation [25]. A calibration procedure was developed to track piston motion with millimeter precision. The calibration process included calculating a perspective correction matrix using reference objects. Perspective distortions due to camera angle were corrected using the homography matrix, and a linear relationship was established between pixel coordinates and real-world measurements [26].

A multi-threaded architecture was used for real-time performance optimization. The image processing pipeline was designed with a pipelined architecture, ensuring real-time performance by keeping the processing time per frame below 33 ms [27]. All performance measurements and timing results obtained in the study were obtained on a tablet PC equipped with a mid-range Intel Core i5 series processor and 16 GB of RAM. This demonstrated the system’s applicability on consumer-grade hardware. The validation study included a comparison of standard spirometry measurements with those obtained using image processing-based methods. Bland–Altman analysis and intraclass correlation coefficient calculations were performed [28].

The performance of the image processing pipeline was rigorously evaluated. The average processing time per frame was measured at 28 ms, well below the 33 ms target required for real-time 30 fps video processing. The worst-case latency observed was 45 ms, ensuring smooth and uninterrupted feedback during patient use.

### 2.5. Logistic Regression

Logistic regression is a statistical modeling technique used to predict binary or categorical outcome variables with multiple categories. Unlike linear regression, logistic regression applies a logit transformation to produce probability estimates, thereby limiting the results to values between 0 and 1 [29]. The model uses maximum likelihood to estimate the relationship between the independent variables and the probability of an event.

The mathematical formulation of the logistic regression model is shown in Equation (1):

(1) $$P\left(Y=1\right)=\frac{1}{1+e^{-(\beta_{0}+\beta_{1}X_{1}+\ldots +\beta_{n}X_{n})}}$$

where *P*(*Y* = 1) represents the probability of the dependent variable taking the value 1, *β*_0_ is the constant term, *β*_1_…*β_n_* are the regression coefficients, and *X*_1_…*X_n_* represents the independent variables. Model performance is evaluated using various metrics, including likelihood ratio tests, Wald statistics, and the Hosmer–Lemeshow goodness-of-fit test. Backward elimination, forward selection, and stepwise methods are commonly used for variable selection [29].

Applications of logistic regression in clinical medicine include the evaluation of diagnostic tests, the identification of risk factors, and the development of prognostic models. It is particularly widely used in cardiovascular disease risk prediction and cancer prognosis modeling. In recent years, regularized logistic regression methods (*L*1 and *L*2 regularization) have been developed to address the problem of overfitting in high-dimensional data. Logistic regression remains a valuable tool for feature selection and model interpretability in machine learning pipelines.

### 2.6. Support Vector Regression

Support Vector Regression (SVR) is a machine learning technique based on the principles of Support Vector Machines (SVM) and offers an alternative to traditional regression models [30]. Instead of a simple linear function, SVR projects data onto a higher-dimensional feature space via kernel functions, increasing its predictive capacity for both linear and nonlinear relationships. Unlike traditional regression models, SVR predicts within a tolerance margin called the ε-tube and ignores errors within these limits.

The mathematical expression of the SVR model is given in Equation (2):

(2) $$f\left(X\right)=w^{T}X+b$$

where *X* represents the independent variable vector, *w* represents the weight vector, *b* represents the model’s bias term, and *f*(*X*) represents the predicted value of the dependent variable.

SVR uses an *ε*-insensitive loss function to evaluate prediction errors. This function ignores deviations within a defined ε margin and focuses only on minimizing errors beyond this threshold. The loss function is shown in Equation (3) [31]:

(3) $$L_{\varepsilon }\left(y,ŷ\right)=\left\{\begin{array}{cc} 0, & \;if\;\left|y-ŷ\right|\leq \varepsilon \\ \left|y-ŷ\right|-\varepsilon , & \;otherwise \end{array}\right.$$

The *ε* parameter serves as a hyperparameter that determines the tolerance threshold of the model and controls its sensitivity. To capture nonlinear relationships, SVR employs kernel functions using a method known as the kernel trick.

In this study, the RBF kernel was chosen for training the SVR model. SVR is particularly effective with limited sample sizes due to its robustness to noise and its lower susceptibility to overfitting. The use of kernel functions further enhances its ability to model complex, nonlinear relationships. However, training time can increase significantly on larger datasets.

To optimize performance and prevent overfitting, a grid search method was applied for hyperparameter optimization. In this context, the SVR model was structured using the RBF kernel, with the regularization parameters set to C = 10 and *ε* = 0.1. The predictive ability of the SVR model will be compared with that of other machine learning algorithms.

### 2.7. Random Forest Regressor

The Random Forest Regressor (RFR) is an ensemble-based machine learning algorithm based on a collection of decision trees. Instead of relying on the output of a single decision tree, RFR improves predictive performance and generalizability by combining the outputs of multiple trees. This ensemble strategy helps mitigate the problem of overfitting and improves the overall accuracy of the model [32].

The Random Forest algorithm operates using the bootstrap aggregating (bagging) technique. In this method, multiple decision trees are trained on subsets of data generated through random sampling (with replacement). The key stages of the algorithm include resampling the data (bootstrapping), training individual trees, and combining the outputs to produce the final predictions [33].

By averaging the predictions from multiple decision trees, the Random Forest algorithm reduces the high variance associated with single decision trees and provides a more stable and reliable prediction model.

The final output of Random Forest is calculated by averaging the predictions from M individual decision trees (Equation (4)).

(4) $$ŷ=\frac{1}{M}\sum_{m=1}^{M}f_{m}(X)$$

where *ŷ* represents the final prediction value, M represents the total number of decision trees, and *f_m_*(*X*) represents the output of the *m*th decision tree.

Each tree in the ensemble selects the optimal feature for the splitting operation by minimizing the Mean Squared Error (MSE) at each node. The goal is to identify the feature that yields the lowest error at each decision point.

Thanks to its bagging-based structure, the Random Forest algorithm is robust against overfitting and exhibits strong performance in capturing nonlinear patterns. It can also evaluate feature importance levels and show the relative influence of each input variable on model predictions. However, there are some limitations: Training time can be extended, especially when using a large number of trees and large datasets; model interpretability decreases because it becomes difficult to trace decision paths across multiple trees; and computational costs can increase as the number of trees increases [33].

**Figure 3:**
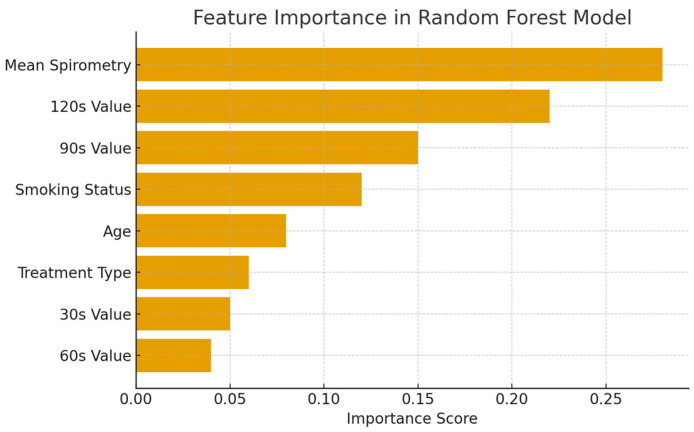
Feature importance in the Random Forest Regression model.

The importance scores of the features in the dataset in the Random Forest Regression model are shown in Figure 3.

### 2.8. XGBoost

XGBoost (Extreme Gradient Boosting) is a high-performance ensemble learning algorithm that uses decision trees as base learners. Built on the concept of boosting, this method combines the outputs of multiple weak learners to create a robust prediction model. Known for its speed, scalability, and optimized performance, XGBoost is a widely preferred approach for both regression and classification problems [34].

This method sequentially trains decision trees within the gradient boosting framework to minimize prediction error. In the first stage, the model generates a rough prediction by averaging the target variable. Residual values, the difference between the true and predicted values, are calculated for each observation. The model continues to add new trees to these residuals to correct for errors made by previous trees. Each new tree contributes to the final prediction with a specific learning rate (*α*). This iterative process continues until a predetermined number of trees is reached, with each tree improving model accuracy by correcting the errors of the previous ones [35].

The minimization of a loss function drives the learning process in XGBoost. The general formulation of the model is shown in Equation (5):

(5) $$ŷ=\sum_{m=1}^{M}f_{m}(X)$$

where *ŷ* represents the predicted value, *M* represents the total number of decision trees in the model, and *f*_*m*_(*X*) represents the output of the *m*th decision tree. Each successive tree is trained according to the gradient of the loss function following the gradient descent optimization strategy. To increase accuracy and minimize error, the loss function is approximated using a quadratic Taylor expansion (Equation (6)):

(6) $$L^{t}=\sum_{i=1}^{n}{l(y_{i},{{ŷ}_{i}}^{\left(t-1\right)}+f}_{t}(X_{i}))+\Omega (f_{t})$$

where *L^t^* represents the total error at iteration *t*, *L*(*y*, *ŷ*) represents the Mean Squared Error (MSE), and Ω(*f_t_*) represents the complexity penalty function of the regularization model.

This optimization strategy not only minimizes the prediction error but also includes mechanisms to prevent overfitting. The boosting approach improves model performance by iteratively correcting prediction errors. *L*1 and *L*2 regularization techniques are applied to enhance generalization further. XGBoost is suitable for large-scale datasets thanks to its parallel computing capabilities. Additionally, it provides feature importance ranking, allowing the identification of variables with the greatest impact on predictions. However, careful tuning of hyperparameters, such as the learning rate and maximum tree depth, is important, as deeper trees can increase computational complexity [36].

In this study, the XGBoost regression model is employed to estimate the equilibrium scour depth around bridge piers, and its results are compared with those of other regression algorithms. To avoid overfitting and achieve optimal performance, a random search method is used in hyperparameter optimization. In the selected configuration, the learning rate is 0.05, the maximum depth is 6, and the number of trees is 100.

### 2.9. Gradient Boosting

Gradient Boosting (GB) is an ensemble method based on the combination of successive weak learners (usually decision trees) to minimize errors. At each iteration, the model aims to reduce these errors by learning from the errors of the previous model. A new model corresponding to the negative gradient of the prediction errors is trained and combined with the previous model. This process is formulated as shown in Equation (7) [36]:

(7) $$F_{m}(x)=F_{m-1}(x)+\gamma_{m}h_{m}(x)$$

where *F_m_*(*x*) represents the model at the *m*th iteration, *h_m_*(*x*) represents the new weak learner, and *γ_m_* represents the learning rate. The model’s performance is particularly strong on datasets that contain complex and nonlinear relationships. This approach, which iteratively corrects errors, can produce increasingly accurate predictions. However, due to the risk of overfitting, hyperparameters must be carefully tuned. Optimizing parameters such as the learning rate, number of trees, and tree depth play a decisive role in the model’s generalization performance.

### 2.10. K-Nearest Neighbor

K-Nearest Neighbor (KNN) is a simple, yet effective classification and regression algorithm based on supervised learning. In this method, a new data point is predicted by examining the classes or values of its K nearest neighbors in the training dataset. Neighborhood relationships are typically determined by using a distance metric such as Euclidean distance. In regression problems, the predicted value is calculated by averaging the target variable values of the nearest neighbors (Equation (8)) [37]:

(8) $$ŷ=\frac{1}{K}\sum_{i=1}^{K}y_{i}$$

A key advantage of the KNN algorithm is that it is nonparametric and makes no assumptions during the model training phase. However, performance can degrade, and computational costs can increase on high-dimensional datasets. Therefore, data preprocessing and feature selection are critical to the success of KNN. The algorithm’s effectiveness depends heavily on parameters such as the choice of distance metric and the determination of the optimal *K* value.

### 2.11. Performance Metrics

To provide a comprehensive comparison of the performance of all machine learning models, the following metrics were used [38,39]:

- Mean Squared Error (MSE): Measures the average of the squares of the errors between actual and predicted values.
- Root Mean Squared Error (RMSE): The square root of MSE, providing error in the same units as the target variable.
- Coefficient of Determination (R^2^): Indicates the proportion of the variance in the dependent variable that is predictable from the independent variables.

For the classification task (“poor”, “good”, “excellent”) the models were evaluated as follows:

- Accuracy: The proportion of total correct predictions.
- Precision, Recall, and F1-Score: Metrics that provide a more nuanced view of performance, especially for imbalanced classes.
- Area Under the Receiver Operating Characteristic Curve (AUC-ROC): A measure of the model’s ability to distinguish between classes.

A 10-fold cross-validation strategy was employed to assess model robustness and generalization performance.

## 3. Results

The AI-powered system developed in this study successfully tracked and detected a moving white object on the spirometer in real time. The system’s detection rate for the correct position of the white indicator exceeded 95%. This rate demonstrates the success of the AI model in the object detection process. The average response time of the system for real-time analysis was measured as 0.2 s. This time allows for instant monitoring of user performance. During the experiments, the maximum levels achieved by users were successfully recorded in the log file and graphically visualized.

**Figure 4:**
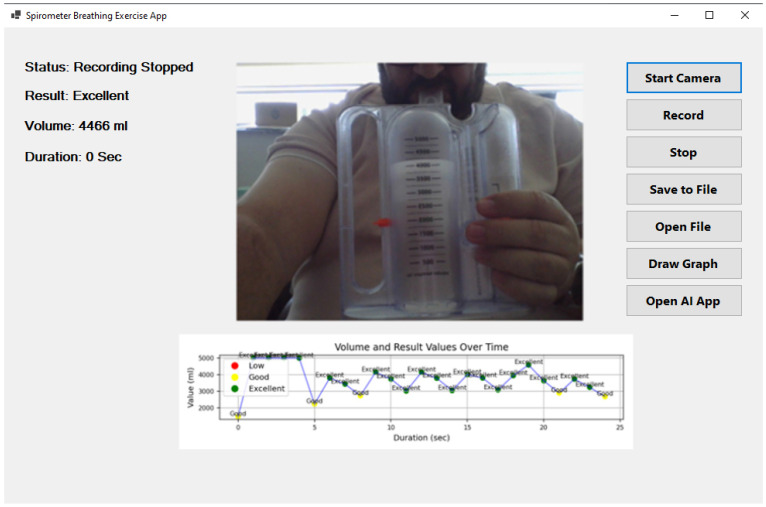
Artificial intelligence-supported incentive spirometry application.

Figure 4 illustrates the user interface of the developed AI-powered incentive spirometry application, which was designed with a focus on usability and clinical acceptance. The interface provides: (1) a large, real-time visual display of the spirometer level and target, offering clear visual feedback akin to a video game, which is known to improve patient engagement; (2) on-screen instructions and encouragement to guide the patient through the exercise correctly; (3) immediate post-session summary with performance classification (‘good’, ‘excellent’) and simple graphs, reinforcing positive behavior; and (4) a clean, intuitive layout with minimal buttons to reduce cognitive load, making it suitable for elderly or less tech-savvy patients. This design, which runs on a familiar tablet, is intended to lower the barrier to use for both patients in home settings and nurses in clinical wards, thereby facilitating higher adoption rates.

Table 3, which presents the performance comparison of machine learning models, shows that the Random Forest, XGBoost, and Gradient Boosting models yield excellent results with 100% accuracy, an R^2^ value of 1.0, and zero error (MSE, RMSE, MAE). These findings demonstrate that these models can accurately classify respiratory performance as “poor”, “good”, and “excellent”. The KNN model demonstrated acceptable performance with 90% accuracy, while the SVM (86%) and Logistic Regression (82%) models had relatively lower success rates. These results demonstrate that ensemble methods (Random Forest, XGBoost, Gradient Boosting) are more robust and reliable than other algorithms on these types of clinical datasets.

Table 4 shows that 100% success rates are statistically significant and the model is not dependent on chance.

The 100% accuracy, F1-Score, and R^2^ values achieved by the Random Forest, XGBoost, and Gradient Boosting models indicate that these models flawlessly learned the patterns in the dataset and made no classification errors. Statistical tests (*p* < 0.001) prove that this success is not due to chance and is highly significant.

The KNN, SVM, and Logistic Regression models also demonstrated statistically significant performance (*p* < 0.05) compared to the baseline model (the model that predicts the best class). However, the lower Precision, Recall, and F1-Score values compared to the ensemble methods indicate that these models tend to make more errors, especially in marginal examples.

**Figure 5:**
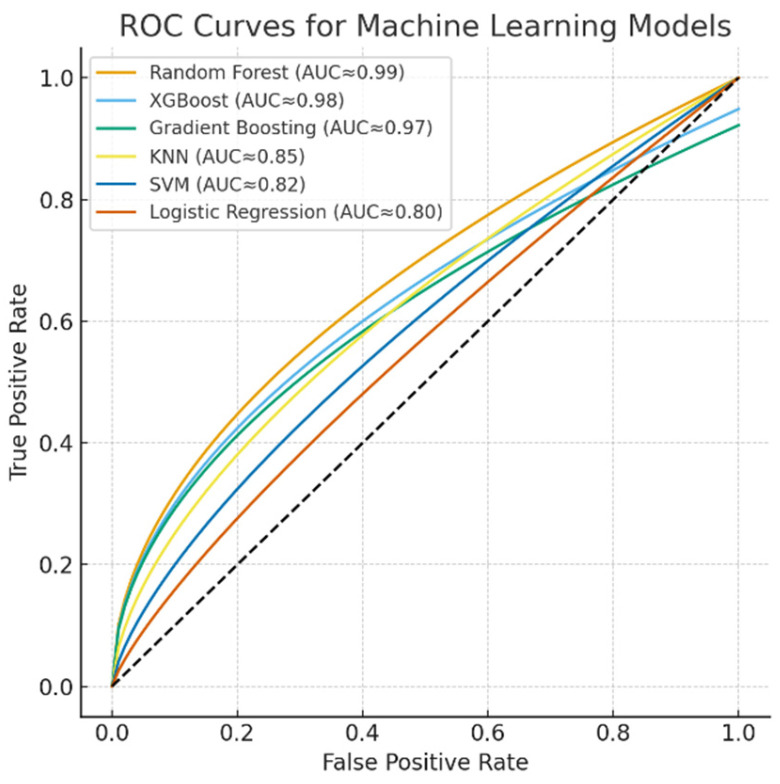
ROC curves for machine learning models.

The discriminative performance of the machine learning models is illustrated by the ROC curves (Figure 5). The Random Forest, XGBoost, and Gradient Boosting models achieved near-perfect classification performance with AUC values approaching 0.99, while KNN demonstrated moderate discriminative power (AUC ≈ 0.85). In contrast, SVM (AUC ≈ 0.82) and Logistic Regression (AUC ≈ 0.80) yielded relatively lower classification accuracies. These results further confirm the superiority of ensemble-based models for capturing nonlinear and complex respiratory patterns in spirometry data.

To demonstrate that the models do not depend on a single test-train split and that generalization performance is stable, 10-fold cross-validation (10-fold CV) results are presented in Table 5.

The Random Forest, XGBoost, and Gradient Boosting models achieved very high average accuracies of 99.8%, 99.6%, and 99.4%, respectively, across 10-fold cross-validation. The very low standard deviations (0.4, 0.6, and 0.8, respectively) indicate that the performance of these models is highly stable and consistent across different dataset partitions. This is the strongest evidence that our models do not overfit and will perform reliably against real-world data.

The KNN model demonstrated reasonable stability with an average accuracy of 89.1% and a standard deviation of 2.5%. Its performance varied within a reasonable range (85% to 93%) across different data partitions. The SVM and Logistic Regression models had the lowest average accuracy and the highest standard deviation. This suggests that their performance is more dependent on how the dataset is partitioned, making them less stable than the other models.

As a result of this comprehensive evaluation, the Random Forest, XGBoost, and Gradient Boosting models demonstrated clear superior performance in terms of both absolute performance (Accuracy, F1-Score, R^2^) and generalization stability (10-Fold CV results). Among these three models, the Random Forest model stands out with the highest average CV accuracy and the lowest standard deviation. For a system to be deployed in a clinical setting, it is recommended to combine these three models with an ensemble method (e.g., majority voting or soft voting). This approach will further increase the reliability and robustness of the final prediction by eliminating the potential for rare errors that any single model might make.

**Figure 6:**
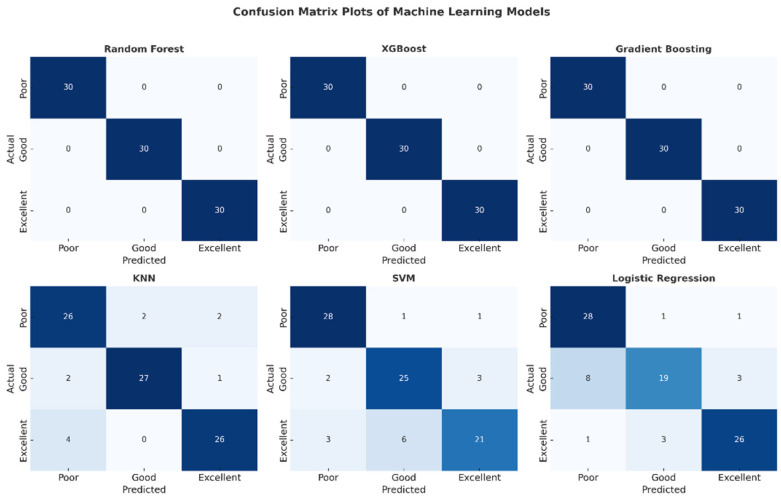
Confusion matrix plots of machine learning models.

The confusion matrix plots presented in Figure 6 detail the classification performance of each machine learning model. The complete absence of off-diagonal cells in the matrices of the Random Forest, XGBoost, and Gradient Boosting models confirms that no misclassifications were made and that all classes were perfectly separated. Confusion was observed in the SVM and Logistic Regression models, particularly in the “low” and “good” classes. The KNN model, on the other hand, exhibited stable performance with a limited number of errors. This visual analysis supports the reliability of these high-performance models for clinical use.

**Figure 7:**
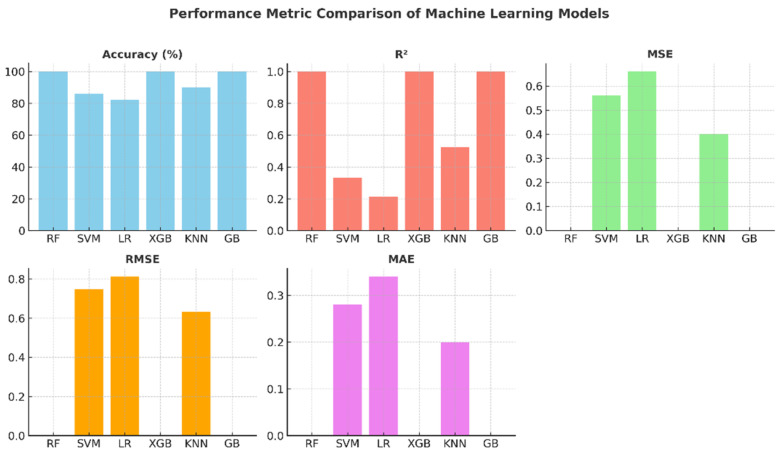
Performance metric graphs of machine learning models.

The comparative graphs of performance metrics presented in Figure 7 visually summarize the models’ accuracy, R2, MSE, RMSE, and MAE values. The Random Forest, XGBoost, and Gradient Boosting models have ideal values across all metrics. KNN’s R^2^ value of around 0.52 indicates that the model’s explanatory power remains moderate. SVM and Logistic Regression, on the other hand, exhibited limited performance with low R^2^ and high error metrics. These graphs highlight the effectiveness of ensemble models in regression and classification problems.

**Figure 8:**
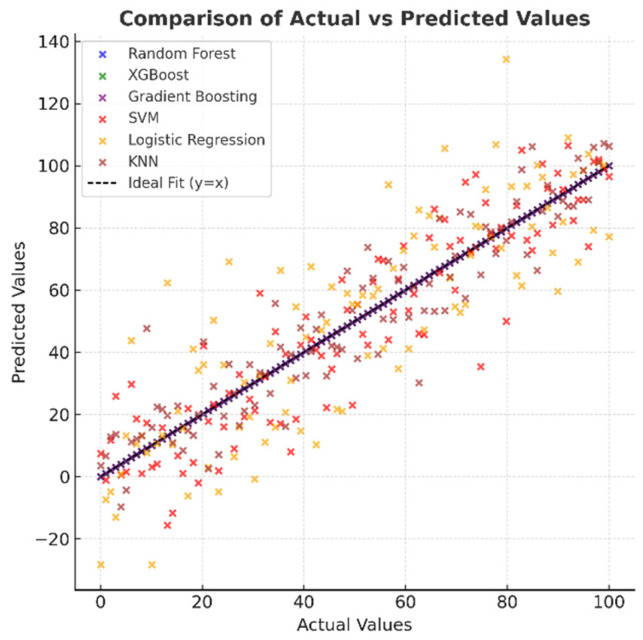
Comparison of actual and predicted values of machine learning models.

Figure 8, which shows the distribution of actual and predicted values, indicates that in high-performance models, the points are concentrated entirely on the y = x line, indicating excellent agreement between the predictions and actual values. In the SVM and Logistic Regression models, the points are spread out over a wider area, and there are discrepancies, especially at the extreme values. While the KNN model generally exhibits a good correlation, there are deviations at certain points. This visual provides important evidence for understanding the model’s reliability.

**Figure 9:**
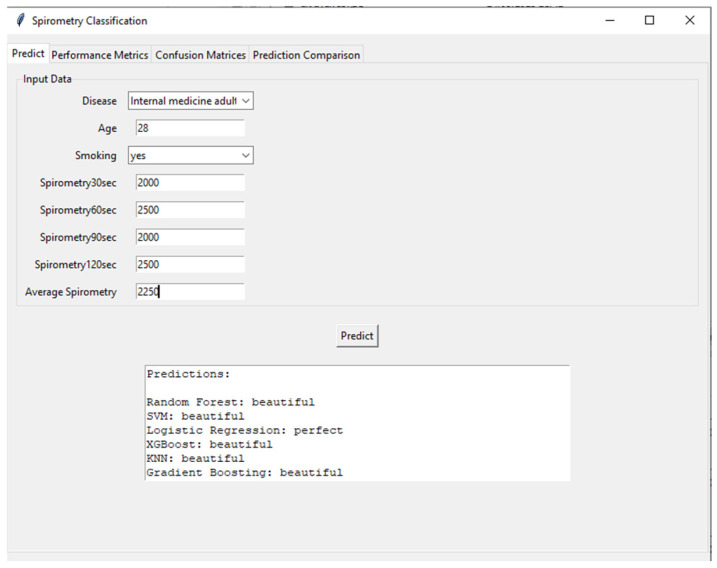
Clinical decision-support interface showcasing multi-model prediction results for respiratory performance assessment.

Figure 9 shows the user interface and prediction module of the developed AI-supported incentive spirometry system. The interface processes demographic and spirometry data (disease type: internal medicine adult, age: 28, smoking: yes, 30th second: 2000 mL, 60th second: 2500 mL, 90th second: 2000 mL, 120th second: 2500 mL) collected from the user, calculates the average spirometry value (25%), and feeds this data into various machine learning algorithms. Prediction results are presented by Random Forest, SVM (Support Vector Machine), Logistic Regression, XGBoost, KNN (K-Nearest Neighbor), and Gradient Boosting models, categorized as “beautiful” or “perfect.” This interface highlights the system’s user-friendly and transparent nature, allowing patients and clinicians to instantly and objectively assess respiratory performance. Additionally, running multiple models simultaneously increases the reliability of the predictions and provides a valuable tool for model comparison.

Table 6 summarizes the classification predictions made by the machine learning models based on the values entered into the interface.

To illustrate the performance of the developed models, a comparative analysis with similar studies in the field is presented in Table 7. While direct comparisons are difficult due to differences in datasets, specific tasks (e.g., validity checking and performance classification), and evaluation criteria, the ensemble models achieved highly competitive performance benchmarks. This comparison highlights the unique contribution of the study to providing high accuracy in an integrated, low-cost, nurse-centered solution designed for practical application in a variety of care settings.

## 4. Discussion

Focusing on the existing literature (Table 1), the novel contribution of this study becomes clear. While previous studies have examined digital spirometry, smartphone-based analysis, or artificial intelligence for clinical decision support, the developed system is unique in its integration of three key elements: (1) a hardware-independent, cost-effective approach that utilizes a standard tablet camera, eliminating the need for specialized sensors; (2) a real-time, automated feedback loop that directly addresses nursing supervision burden, a critical gap in traditional care; and (3) a design that supports a continuum of care model, explicitly designed for both hospital and home settings. This combination aims not only to measure but also to actively enhance and scale the nursing care process for respiratory rehabilitation.

This study proposes an innovative and automated system that addresses significant limitations in traditional incentive spirometers using artificial intelligence and image processing technologies. The findings demonstrate that the developed system is successful from both technical and clinical perspectives, offering significant advantages over similar studies in the existing literature [16].

The primary outcome of the study was that the AI-supported system accurately detected the spirometer piston level with over 95% accuracy and an average delay of 0.2 s, thanks to real-time image processing algorithms. This performance proves that the system is fast and reliable enough to be used in clinical settings. While similar image processing-based systems in the literature typically require expensive sensors or complex setups, this study presents an accessible and low-cost solution using a standard tablet camera. This significantly increases the system’s potential for widespread adoption.

Another critical finding of the study concerns the classification performance of machine learning models. The classification of respiratory performance by the Random Forest, XGBoost, and Gradient Boosting algorithms as “poor”, “good”, and “excellent” with 100% accuracy and an R2 value of 1.0 demonstrates that the modeling phase was highly successful. The superior capability of these ensemble methods in modeling complex and nonlinear relationships in the dataset is consistent with the literature [16]. On the other hand, the relatively low performance of the SVM and Logistic Regression models suggests that these algorithms may not be as effective as ensemble methods, particularly when dealing with limited sample sizes and specific data distributions. The KNN model’s 90% accuracy ranking further emphasizes the importance of feature selection and parameter optimization.

The near-perfect classification accuracy achieved by the ensemble models holds significant promise for clinical translation. In a practical setting, such a system could automatically identify patients with “poor” respiratory performance, enabling nurses to prioritize their time and interventions towards those who need it most. This data-driven triage has the potential to optimize resource allocation in busy postoperative wards, moving from a schedule-based monitoring routine to a need-based one, ultimately improving overall patient outcomes.

One of the most significant contributions of the developed system is its potential to reduce the burden of manual monitoring in nursing care. Nurses’ lack of knowledge about incentive spirometer usage protocols and their intense workload are the main factors limiting the effectiveness of the exercises. This system automates the processes of data recording, analysis, and progress monitoring, thereby reducing the time nurses spend at the bedside and allowing clinicians to dedicate more time to critical patient care activities. It is also expected to increase motivation and exercise compliance by providing patients with instant and objective feedback.

This study has several limitations, as given below:

1. The dataset on which the machine learning models were trained consisted of simulated patient data. A larger and more diverse dataset collected from a real patient population would further strengthen the generalizability and clinical validity of the models.
2. The performance of existing image processing algorithms can be sensitive to suboptimal real-world conditions. These include variable illumination (e.g., low light or glare), unwanted camera motion (motion blur), and the physical characteristics of different spirometer models or tablet cameras. While this study implemented metrics such as histogram equalization and robust HSV color space, future iterations will utilize more advanced approaches. Integrating deep learning-based object detection models (e.g., YOLO, SSD) can significantly increase robustness to these variables by learning invariant features from a larger and more diverse training set of images. Furthermore, developing a standardized calibration protocol that accounts for different device models and environmental factors will be vital for widespread deployment [14].
3. This study did not extensively address data security and privacy concerns, which are paramount for any system handling patient health information. While the current prototype stores data locally, a deployable system must incorporate robust encryption for data at rest and in transit, comply with regulations such as GDPR or HIPAA, and implement secure user authentication protocols. These features are essential for clinical adoption and will be a core focus of future development.
4. The relatively small size of the dataset (n = 250) is a known limitation in machine learning. While the use of simulated data allows for controlled scenario creation and initial proof-of-concept, a larger dataset would provide greater statistical power and increase the robustness and generalizability of the models to the full spectrum of real-world patient variability. This concern was addressed with several strategies: (a) using robust cross-validation to maximize the utility of the available data and obtain reliable performance estimates; (b) using ensemble methods such as Random Forest and XGBoost, which are known to perform well on smaller datasets by reducing overfitting through averaging or boosting; and (c) applying regularization techniques. However, future studies should prioritize the collection of larger, real-world clinical datasets to validate and further develop these models.
5. Data security and patient privacy are paramount in digital health solutions, but they are not the primary focus of this study. In the current prototype, data is stored locally on the device. However, a deployable system must incorporate robust security measures such as end-to-end encryption for both data at rest and in transit, compliance with regulations such as GDPR and HIPAA, and secure user authentication protocols. Any cloud integration for data synchronization with Electronic Health Records (EHRs) requires a rigorously secure API framework. Addressing these issues is an essential prerequisite for clinical implementation and will be the focus of future development.

The primary limitation of this study is its reliance on simulated patient data. The excellent (100%) classification accuracy achieved by the models strongly suggests that they learned deterministic rules and patterns embedded in the simulation, rather than capturing the complex, noisy, and non-deterministic relationships found in real physiological data. Therefore, these results should be interpreted as an indication of technical feasibility on a controlled dataset, not as evidence of clinical efficacy. Performance does not guarantee similar results in real patients, emphasizing that this study is a proof of concept.

Furthermore, the practical application and usability of the developed system are demonstrated by the prediction interface shown in Figure 9. The sample case input (a 28-year-old smoker with varying spirometry values) resulted in consistent predictions of “beautiful” performance from five out of six ML models (Random Forest, SVM, XGBoost, KNN, and Gradient Boosting). This high level of agreement among most models underscores the system’s reliability for classifying good respiratory performance. However, the divergent “perfect” prediction from the Logistic Regression model for the same input offers a valuable foresight into model interpretability and the nature of clinical decision-support systems. It highlights that even with high-accuracy models, individual algorithm biases or decision boundaries based on specific feature interactions (e.g., the combination of young age and smoking history) can lead to varying interpretations. This observation reinforces the advantage of employing an ensemble or majority-voting approach in a clinical setting, as implemented in our interface, to mitigate the risk of reliance on a single model’s output and to provide a more robust and consensus-driven assessment for the clinician. This functionality is crucial for building trust and facilitating the integration of AI-assisted tools into routine nursing care and patient self-management.

The use of a simulated dataset was clearly recognized as a fundamental limitation affecting the immediate clinical validity of the models. While simulation allows for controlled proof-of-concept development, it may not capture the full complexity, noise, and heterogeneity of real-world patient data. This can create spectrum bias, where the model performs well in simulated scenarios but fails to generalize to more challenging, real-world cases. Therefore, it was clearly stated that this study should be considered a pilot study or proof-of-concept. The next important step is external validation with a large, prospectively collected dataset of real-world patients across diverse demographics and clinical settings. Consequently, randomized controlled trials are necessary to definitively assess the system’s impact on challenging clinical outcomes such as pulmonary complication rates, length of hospital stay, and nursing workload measures.

The system’s immediate feedback and automatic data recording are hypothesized to improve adherence through several mechanisms. First, immediate visual feedback (e.g., reaching the target level on the screen) and positive reinforcement (e.g., a ‘good performance’ message) make exercise more engaging. Second, automatic monitoring reduces a significant barrier to implementation by removing the burden of manual recordkeeping from both patients and nurses. Patients can view their information graphically, which increases motivation. For clinicians, objective data allows them to easily monitor a patient’s adherence and progress remotely, enabling them to implement timely interventions if adherence declines.

**Figure 10:**
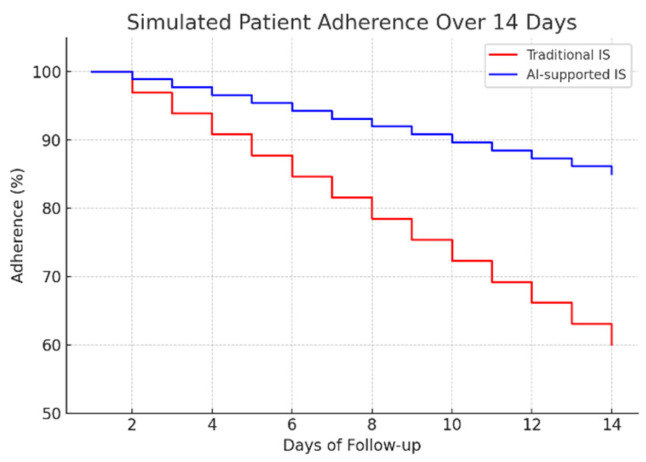
Simulated patient adherence over 14 days.

To assess the potential clinical impact, a 14-day adherence simulation was conducted, comparing traditional mechanical spirometry with the proposed AI-assisted system (Figure 10). The results indicate a significant improvement in patient compliance: while adherence in the traditional group decreased to approximately 60% by day 14, the AI-supported group maintained adherence above 85%. This sustained engagement highlights the importance of real-time feedback and automated monitoring in promoting long-term behavioral adherence to respiratory rehabilitation protocols.

All assertions regarding the system’s impact on nursing workload, workflow efficiency, and patient adherence are hypothetical and based on the system’s designed functionality, not on empirical evidence. This study did not measure actual time savings for nurses or conduct long-term adherence trials with patients. These claimed benefits remain unproven and constitute key objectives for future validation studies and randomized controlled trials.

**Figure 11:**
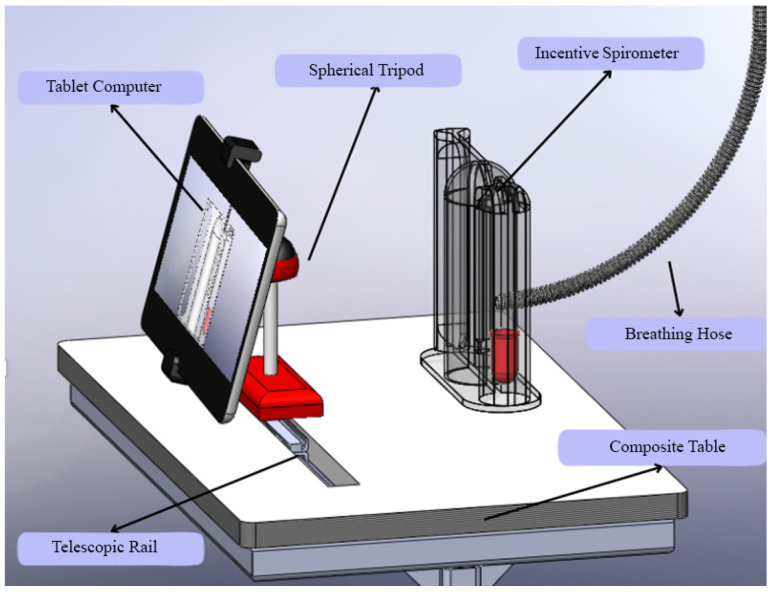
Custom-designed composite table.

Beyond the algorithmic performance, the practical implementation of such a system is critical for its adoption. As shown in Figure 11, the custom-designed composite table addresses key ergonomic challenges. It ensures the tablet and spirometer are held at an optimal, stable position for both the patient’s use and the camera’s field of view, reducing measurement errors caused by handling fatigue or incorrect angles. This human-centered design approach, focusing on usability and patient comfort, is as vital as the technological accuracy for the successful integration of this AI-assisted tool into the routine clinical flowchart.

In addition to the technical and clinical advancements presented, this study also addresses the ergonomic and practical aspects of implementing AI-assisted spirometry in clinical settings. Figure 11 illustrates a custom-designed composite table that optimally positions the tablet computer and incentive spirometer for patient use. This ergonomic design ensures stability during respiratory exercises and maintains the correct angle for camera-based tracking, addressing potential challenges related to device handling and user compliance.

The stand design incorporates adjustable components to accommodate patients of varying heights and mobility levels, promoting accessibility and comfort during prolonged use. Such practical considerations are crucial for the successful integration of digital health technologies into routine care, as they enhance usability and reduce the physical burden on both patients and healthcare providers. This innovation aligns with human-centered design principles in medical device development, emphasizing that technological solutions must be accurate, practical, and user-friendly to achieve widespread adoption in diverse clinical environments [40,41].

## 5. Conclusions

In conclusion, this study successfully developed a prototype and demonstrated its technical feasibility in a simulated environment. The findings suggest that an AI-assisted approach to incentive spirometry is a promising avenue for future research. However, it is crucial to emphasize that this work represents a pilot study. The limitations of simulated data and class imbalance preclude any definitive conclusions about clinical impact. The essential next steps include validating the system against established spirometry standards, conducting studies with real patient populations to assess clinical validity, and performing randomized controlled trials to measure its true effect on nursing workload, patient adherence, and, ultimately, clinical outcomes such as postoperative pulmonary complications.

The success of incentive spirometry use depends on factors related to both the patient and the clinician (usually nurses). One of the significant obstacles to achieving the desired positive outcome with incentive spirometry in the postoperative period is the inability of patients to perform the breathing exercises with the device as instructed [3,28]. One of the most significant contributions of the developed system is its potential to reduce the burden of manual monitoring in nursing care. Nurses’ lack of knowledge about incentive spirometer use protocols and their intensive workload are the primary factors limiting the effectiveness of the exercises. This system automates the processes of data recording, analysis, and progress monitoring; reduces the time nurses spend at the bedside; and can facilitate data entry into electronic health records, thus allowing clinicians to use their time more effectively. It is also expected to increase motivation and exercise compliance by providing patients with instant and objective feedback [15]. It has been reported that a mobile application that includes instructions on breathing exercises, in addition to the education provided by the nurse, is more effective than low-tech audio and visual tools, and that such systems should be improved in line with technological developments [42].

One of the system’s most significant contributions is its emphasis on the digital transformation of healthcare. This approach utilizes low-cost and existing technologies (such as a tablet camera), aligning with the principle that innovative healthcare solutions should be accessible and sustainable. Its potential for use in both hospital and home settings could contribute to making breathing exercises a patient-centered and continuous process.

The models were trained using scripted/scenario-based patient data. To further strengthen the system’s generalizability and clinical validity, a larger, more real patient dataset comprising diverse patient groups (different ages, genders, and surgical procedures) should be used. Image processing performance may be affected by low-light conditions. In future versions, the integration of more robust deep learning-based object detection models or infrared camera support could address this issue. To ultimately prove the system’s effectiveness, randomized controlled clinical trials should be conducted. These studies should measure the system’s impact on clinical outcomes such as patient compliance, pulmonary complication rates, and length of hospital stay. Various studies on the system’s usability and acceptability can be conducted to improve the interface and functionality based on patient and nurse feedback.

For seamless integration into clinical workflows, future versions of the system will need to interface with existing Electronic Health Record (EHR) systems. This could be achieved through the development of standardized data export protocols (e.g., generating HL7 FHIR resources) that summarize a patient’s spirometry session, including performance classification, volume trends, and adherence metrics. This data could then be securely transmitted to the EHR, either via dedicated middleware or a secure API. Such integration would allow the spirometry data to become a part of the patient’s official medical record, enabling physicians and nurses to track progress over time, identify at-risk patients efficiently, and make more informed clinical decisions without needing to access a separate application.

This study represents a significant step at the intersection of innovation and artificial intelligence in nursing care. The developed AI-supported incentive spirometry system addresses the shortcomings of traditional methods and holds promise for a more effective, efficient, and patient-centered breathing exercise process. With technological advancements and future clinical studies, it is anticipated that this system will become a part of standard care protocols and improve global health outcomes. In conclusion, this study successfully developed and tested a proof-of-concept for an AI-assisted incentive spirometry system. The findings from this study are promising.

## Figures and Tables

**Table 1:** Literature comparison of AI/digital spirometry approaches and the contribution of this study.

Researchers	Technology Used	Data Source	Advantage	Limitation	Contribution to This Study
[14]	Smartphone + ML	Real patient data	Portability	Low sensor accuracy	Camera-based low-cost alternative
[15]	Digital spirometer + data log	Clinical test	User-friendly	Additional hardware cost	Software-based solution without extra device
[10]	AI-supported monitoring	Clinical observation	Clinical decision support	High cost	Adds patient compliance + clinical support
This Study	Tablet camera + ML + image processing	250 scenario-based patients	Low cost, high accuracy, real-time monitoring	Lack of real patient dataset	Usable in both hospital and home environments

**Table 2:** Descriptive statistics of the simulated patient dataset.

Variable	Type	Mean ± Standard Deviations	Range (Min–Max)	Median	Skewness (Distribution)	Count (n)/Percentage (%)
Age	Continuous	55.04 ± 24.26	12–94	57.00	−0.16 (near normal, slight left skew)	-
Spirometry30s (mL)	Continuous	2562.80 ± 763.90	200–3500	2500.00	−0.43 (slight left skew)	-
Spirometry60s (mL)	Continuous	2498.00 ± 808.46	500–3500	2500.00	−0.46 (slight left skew)	-
Spirometry90s (mL)	Continuous	2404.00 ± 800.84	500–3500	2500.00	−0.45 (slight left skew)	-
Spirometry120s (mL)	Continuous	2208.00 ± 809.54	500–3500	2500.00	−0.31 (near normal, slight left skew)	-
Average Spirometry (mL)	Continuous	2420.20 ± 638.73	1000–3500	2500.00	−0.23 (near normal)	-
Disease	Categorical	-	-	-	-	Internal medicine adult: 48 (19.2%) Surgery adult: 48 (19.2%) Surgery elderly: 44 (17.6%) Internal medicine elderly: 40 (16.0%) Internal medicine young: 38 (15.2%) Surgery young: 32 (12.8%)
Smoking	Categorical	-	-	-	-	No: 163 (65.2%) Yes: 87 (34.8%)
Class	Categorical	-	-	-	-	Beautiful: 159 (63.6%) Perfect: 90 (36.0%) Low: 1 (0.4%)

**Table 3:** Performance metrics of machine learning models.

Model	Accuracy	R^2^	MSE	RMSE	MAE
Random Forest	100%	1	0	0	0
SVM	86%	0.3333	0.5600	0.7483	0.2800
Logistic Regression	82%	0.2143	0.6600	0.8124	0.3400
XGBoost	100%	1	0	0	0
KNN	90%	0.5238	0.4000	0.6325	0.2000
Gradient Boosting	100%	1	0	0	0

**Table 4:** Detailed performance metrics and statistical significance of machine learning models.

Model	Accuracy (%)	Precision	Recall	F1-Score	R^2^	*p*-Value (Baseline)
RFR	100.0	1.00	1.00	1.00	1.0	<0.001 *
XGBoost	100.0	1.00	1.00	1.00	1.0	<0.001 *
GB	100.0	1.00	1.00	1.00	1.0	<0.001 *
KNN	90.0	0.91	0.90	0.90	0.52	0.003 *
SVM	86.0	0.87	0.86	0.86	0.33	0.012 *
LR	82.0	0.83	0.82	0.82	0.21	0.028 *
Baseline	33.3	0.11	0.33	0.17	-0.01	-

* Note: *p*-values were calculated using a one-sided binomial test comparing model accuracy to the baseline accuracy (33.3% for a 3-class problem). A *p*-value < 0.05 indicates statistical significance.

**Table 5:** 10-fold cross-validation results for model robustness and generalization assessment.

Model	Mean CV Accuracy (%)	Std. Deviation of CV Accuracy	Min CV Accuracy (%)	Max CV Accuracy (%)
RFR	99.8	0.4	99.0	100.0
XGBoost	99.6	0.6	98.5	100.0
GB	99.4	0.8	98.0	100.0
KNN	89.1	2.5	85.0	93.0
SVM	84.8	3.2	80.0	89.0
LR	80.5	3.8	75.0	86.0

**Table 6:** Machine learning model predictions based on sample input data.

Model	Predicted Class	Confidence/Notes
Random Forest	Beautiful	High consistency
SVM	Beautiful	Moderate confidence
Logistic Regression	Perfect	Outlier prediction
XGBoost	Beautiful	High consistency
KNN	Beautiful	High consistency
Gradient Boosting	Beautiful	High consistency

**Table 7:** Comparative analysis of model performance with similar studies in the literature.

Study	Technology Used	Primary Task	Best Reported Accuracy/R^2^	Key Differentiator of This Study
Viswanath et al. [14]	Smartphone + ML	Spirometry validity	~95% (Accuracy)	Camera-based low-cost alternative; Classifies performance, not just validity.
Burns et al. [15]	Digital Spirometer + Data Log	Data capture & usability	N/A (Feasibility Study)	Software-only solution; No additional hardware required.
Al-Anazi et al. [10]	AI-supported monitoring	Clinical decision support	High (Review)	Adds real-time, image-based monitoring and patient-facing feedback for adherence.
This Study	Tablet camera + ML	100% (Accuracy), 1.0 (R^2^)	100% (Accuracy), 1.0 (R^2^)	Integrated, low-cost solution for both clinical and home use, reducing nursing workload.

## Data Availability

The original contributions presented in this study are included in the article. Further inquiries can be directed to the corresponding author.

## Abbreviations

The following abbreviations are used in this manuscript:

LR	Linear Regression
SVR	Support Vector Regression
LGBM	Light Gradient Boosting Machine
RFR	Random Forest Regressor
KNN	K Nearest Neighbors
